# Gradual Rewarming with Gradual Increase in Pressure during Machine Perfusion after Cold Static Preservation Reduces Kidney Ischemia Reperfusion Injury

**DOI:** 10.1371/journal.pone.0143859

**Published:** 2015-12-02

**Authors:** Paria Mahboub, Petra Ottens, Marc Seelen, Nails t Hart, Harry Van Goor, Rutger Ploeg, Paulo Martins, Henri Leuvenink

**Affiliations:** 1 Dept of Surgery, Groningen Transplant Center, University Medical Center Groningen, University of Groningen, Groningen, The Netherlands; 2 Dept of Internal Medicine, Division of Nephrology, University Medical Center Groningen, University of Groningen, Groningen, The Netherlands; 3 Dept of Pathology and Medical Biology, University Medical Center Groningen, University of Groningen, Groningen, The Netherlands; 4 Nuffield Department of Surgical Sciences, University of Oxford, Oxford, United Kingdom; 5 Dept. of Surgery, Division of Transplantation, University of Massachusetts, Worcester, MA, United States of America; University of Torino, ITALY

## Abstract

In this study we evaluated whether gradual rewarming after the period of cold ischemia would improve organ quality in an Isolated Perfused Kidney Model. Left rat kidneys were statically cold stored in University of Wisconsin solution for 24 hours at 4°C. After cold storage kidneys were rewarmed in one of three ways: perfusion at body temperature (38°C), or rewarmed gradually from 10°C to 38°C with stabilization at 10°C for 30 min and rewarmed gradually from 10°C to 38°C with stabilization at 25°C for 30 min. In the gradual rewarming groups the pressure was increased stepwise to 40 mmHg at 10°C and 70 mmHg at 25°C to counteract for vasodilatation leading to low perfusate flows. Renal function parameters and injury biomarkers were measured in perfusate and urine samples. Increases in injury biomarkers such as aspartate transaminase and lactate dehydrogenase in the perfusate were lower in the gradual rewarming groups versus the control group. Sodium re-absorption was improved in the gradual rewarming groups and reached significance in the 25°C group after ninety minutes of perfusion. HSP-70, ICAM-1, VCAM-1 mRNA expressions were decreased in the 10°C and 25°C groups. Based on the data kidneys that underwent gradual rewarming suffered less renal parenchymal, tubular injury and showed better endothelial preservation. Renal function improved in the gradual rewarming groups versus the control group.

## Introduction

Current preservation in organ transplantation is based on hypothermic preservation. The standard practice is to preserve organs by static cold storage (SCS) at 4°C until the time of implantation. Although metabolism is reduced during hypothermia, it is not completely arrested. Even at 4°C, cells continue to consume oxygen and utilize ATP at a metabolic rate of approximately 5% of baseline.[1,2] This leads to a gradual depletion of ATP and ADP, which stops almost all energy-dependent processes and also initiates early damage. All these factors contribute to cold ischemia injury in the organ during static cold preservation. At the time of reperfusion, graft rewarming and reoxygenation induces even more damage than the initial tissue damage caused by ischemia due to formation of reactive oxygen species.

Alternative preservation approaches to improve graft quality during organ preservation (mainly liver) are currently being studied by many groups. Major developments are machine perfusion methods such as hypothermic, sub-normothermic and even normothermic perfusion. It is shown that a period of hypothermic oxygenated machine perfusion [3,4] or subnormothermic machine perfusion [5] prior to the reperfusion has been beneficial in increasing the ATP content of the graft which later helps to protect the organ against ischemia reperfusion injury.[6,7] Alongside hypothermic and sub-normothermic machine perfusion, normothermic machine perfusion (NMP) has been applied prior to reperfusion. Adding a period of NMP after SCS and before implantation of the organ offers potential to assess graft viability prior to transplantation.[8,9] NMP includes a pulsatile flow of oxygenated perfusion solution in the organ which supports cellular metabolism at body temperature restores the energy content of the organ, and washes out waste products prior to reperfusion in the recipient body. Nicholson and colleagues have shown the benefits of kidney NMP in several studies and the method has been applied in human organs with success.[10,11] Although machine perfusion is associated with better graft function after transplantation and may protect against ischemia reperfusion injury, there has been little attention on strategies to protect the organ from sudden graft rewarming and reoxygenation during machine perfusion.[5]

In this study we investigated whether a strategy of a gradual increase in temperature and pressure after cold storage, prior to reperfusion at body temperature improves kidney graft quality.

## Methods

### Animals used

Male Lewis rats (Harlan, The Netherlands) weighing 290–350 g were used in this study. Animals received care according to the Dutch Law on animal experiments. The study protocol was approved by the Institutional Animal Care and Use Committee of the University of Groningen (IACUC-RuG).

Rats were anesthetized using 5% isoflurane and 1ml 0.9% NaCl with 500 IU of heparin was administrated via the dorsal penile vein. The rats were sacrificed after left nephrectomy. The renal artery and ureter were cannulated. The kidneys were then flushed via the renal artery with 5 ml of cold (4°C) saline (Baxter, The Netherlands) followed by 5 ml of cold (4°C) University of Wisconsin (UW) preservation solution (Viaspan, Belzer ™). The kidneys were cold stored at 4°C for a period of 24 hours in UW in a 25 mL flask. After CS, kidneys were placed in an isolated kidney perfusion (IPK) device.

### The Isolated perfused kidney (IPK) device and perfusion settings

The IPK device consists of a roller pump (Ismatec MS-2/6-160; IDEX Health and Science), heat exchanger (Radnoti Heating coil, 5.5 mL), one tubular membrane oxygenator, 100 mL solution reservoir, an inline temperature probe and pressure probe (Edwards Lifescience Corporation). The device was pressure and temperature controlled. Pressure was monitored continuously by a probe connected to a lap top during the IPK experiment. The heat exchanger was connected to two (one cold and one warm) water baths (Julabo Labortechnik). The organ chamber was covered by a Perspex lid which helped to provide a moist environment for the perfused rat kidney.

The kidneys were placed in the organ chamber and connected to the IPK device and perfused through the renal artery with oxygenated William’s medium E (WME). The ureter was cannulated and the ultra-filtrate (urine) was collected.

### Experimental groups

Following 24 hours of SCS (4°C) kidneys were connected to the IPK device and perfused during 90 minutes according to one of the following protocols.

**Control group (immediate rewarming) (n = 8)**: Kidneys were immediately perfused at 38°C at a mean arterial pressure of 100 mm Hg during 90 minutes perfusion “Table 1”.

**Table 1:** This table illustrates the details of study design including duration of cold storage, perfusion temperature, perfusion pressure, rewarming and reperfusion phase.

Groups n = 8	Cold Storage	Rewarming	Reperfusion
**Control**	24 h	38°C/100mmHg/30 min	38°C/100mmHg/60 min
**10°C**	24 h	10°C/40mmHg/25min 25°C/70 mmHg/5min	38°C/100mmHg/60 min
**25°C**	24 h	10°C/40mmHg/5min 25°C/70 mmHg/25min	38°C/100mmHg/60 min

**Gradual rewarming from 10°C to 38°C (n = 8):** Kidneys were first perfused at a temperature of 10°C for 25 minutes. Afterwards, the temperature was gradually increased to 38°C in two steps. First it was increased to 25°C for a few minutes, and next it was raised to 38°C and perfused at 38°C for additional 60 minutes. Parallel to increasing the temperature, the pressure was gradually elevated from 40 mm Hg to 70 mm Hg at 25°C and to 100 mm Hg at 38°C “Table 1”.

**Gradual rewarming from 25°C to 38°C (n = 8)**: Kidneys were placed in the IPK set-up and the temperature was set on 10°C in the beginning and then gradually raised from 10°C to 25°C and was stabilized at 25°C for 25 minutes. Alongside to this, pressure was increased from 40 mm Hg to 70 mm Hg. After first 30 minutes the temperature was adjusted at 38°C with pressure set to 100 mm Hg for 60 minutes “Table 1”.

**Cold static preservation group (n = 6):** Followed nephrectomy kidneys were subjected to 24 hours SCS in UW solution at 4°C without rewarming. After SCS tissue samples were taken and stored at -80°C in order to measure ATP content.

### Perfusion solution

The perfusion solution consists of William’s Medium E (Life technologies, USA) 100 mL, Creatinine (Sigma-Aldrich, The Netherlands) 0.08 g/dL, Bovine Serum Albumin (PAA Laboratories GmbH, Austria) 5g/dL, HEPES (Sigma-Aldrich, The Netherlands) 0.7149 g/dL. This solution was used for the 90 minutes perfusion period. Prior to the experiments, the perfusion solution was oxygenated during 15 minutes with carbogen (95%O_2_ and 5%CO_2_) to achieve an oxygen pressure of at least 60 kPa and it was kept actively oxygenated. After this equilibration the pH was adjusted to 7.4. During the IPK perfusion no further adjustments were made to the pH.

### Temperature hemodynamic monitoring

Temperature and renal flow were recorded every 10 minutes during the IPK perfusion.

### Perfusate and ultrafiltrate sampling and Analysis

Perfusate samples were collected after 15, 30, 60 and 90 minutes of perfusion and stored at -80°C for further analysis. Ultrafiltrate production was measured at the same time points and the samples were stored at -80°C. Fractional re-absorption of sodium ((perfusate sodium-ultrafiltrate sodium) / (perfusate sodium) ×100) and creatinine clearance (ultrafiltrate creatinine × ultrafiltrate volume/perfusate creatinine) were calculated. Lactate level and arterial pH were measured by an ABL800 FLEX analyzer (Radiometer, Brønshøj, Denmark).

### Renal injury biomarkers

Indicators of renal cellular injury were analyzed in the perfusate and ultrafiltrate.[12,13] Aspartate transaminase (AST) and lactate dehydrogenase (LDH) were measured in the perfusate. N-acetyl-ß-D-glucosamine (NAG) was measured in the ultrafiltrate samples as it is an indicator of ischemic tubular damage in kidney.[14] The methodology for these biochemical analyses has been described in detail previously.[15]

### Lipid peroxidation

Oxygen free radical (OFR) induced injury was measured by the level of lipid peroxidation in the perfusate samples. The methodology has been described previously.[16]

### mRNA expression assay

Details of real-time reverse transcription polymerase chain reaction (qRT-PCR) have been reported previously.[17]

Gene expression of kidney injury molecule-1 (KIM-1), heat shock protein-70 (HSP-70), intercellular adhesion molecule 1 (ICAM-1), vascular cell adhesion molecule-1 (VCAM-1), P-selectin and β-actin (as housekeeping gene) were measured. Based on the mean of β-actin mRNA content, gene expression was normalized and calculated. Results were represented as 2-ΔCT (CT threshold cycle). Primers are listed in “Table 2”.

**Table 2:** qRT PCR primers of the housekeeping gene (β-actin), KIM-1, HSP-70, ICAM-1, VCAM-1 and P-selectin primers and their sequences.

Primers	Forward	Reverse	Amplicon (bp)
**β-actin**	5’-GGAAATCGTGCGTGACATTAAA-3’	5’-GCGGCAGTGGCCATCTC-3’	109
**KIM-1**	5’-AGAGAGAGCAGGACACAGGCTTT-3’	5’-ACCCGTGGTAGTCCCAAACA-3’	89
**HSP-70**	5’-GGTTGCATGTTCTTTGCGTTTA-3’	5’-GGTGGCAGTGCTGAGGTGTT-3’	97
**ICAM-1**	5’-CCAGACCCTGGAGATGGAGAA-3’	AAGCGTCGTTTGTGATCCTCC	251
**VCAM-1**	5’-TCTCTGGGTCTTCGTGTTTCTTATCT-3’	5’-GTGTCCCCCTAGTACCATCTGAA-3’	80
**P-selectin**	5’-TGTGGAAGTGTGCCCGAAA-3’	5’-ACGAGCCATTAACAGACTTTAGCA-3’	84

### Energy store—Adenosine 5-triphosphate (ATP)

Tissue concentration of ATP was used as an indicator of the energy status of the grafts. Kidney samples were taken after cold storage in the reference group and after perfusion in the control, and in the experimental groups. Samples were snap frozen in liquid nitrogen. Frozen tissue was cut into 20μm slices and a total amount of ± 50mg was homogenized in 1 mL of SONOP (0.372g EDTA in 130mL H_2_O and NaOH (pH 10.9) + 370 mL 96% ethanol) and sonificated. The precipitate was removed by centrifugation (13,000 rcf for 10 min). In order to achieve a protein concentration of 200–300 mg/mL (Pierce BCA Protein Assay Kit, Thermo Scientific, Rockford, IL) supernatant was diluted with SONOP and mixed with 450mL of 100mM phosphate buffer (Merck; pH 7.6–8.0). Fifty microliters of phosphate buffered supernatant was used for ATP measurement using ATP Bioluminescence assay kit CLS II (Boehringer, Mannheim, Germany) and a luminometer (Victor^3TM^ 1420 multilabel counter, PerkinElmer). ATP concentrations were calculated from a calibration curve constructed on the same plate, corrected for the amount of protein, and values were expressed as μmol/g protein.

### Histology

Renal tissues were collected at the end of the perfusion and were fixed in 10 percent formalin. The tissue blocks were embedded in paraffin and were cut at 4 μm and stained with the Periodic acid-Schiff (PAS) methods for evaluation using light microscopy. Slides were scored at 4 fields in order to assess changes in morphological parameters by two independent investigators.

### Statistical analysis

The data is represented as mean ± standard deviation. P value is analyzed using Mann-Whitney U test. Analyses is performed using SPSS software version 16.0 (Inc., Chicago, IL, USA). A p-value of less than 0.05 was considered significant.

## Results

### Temperature and hemodynamic monitoring

**Fig 1:**
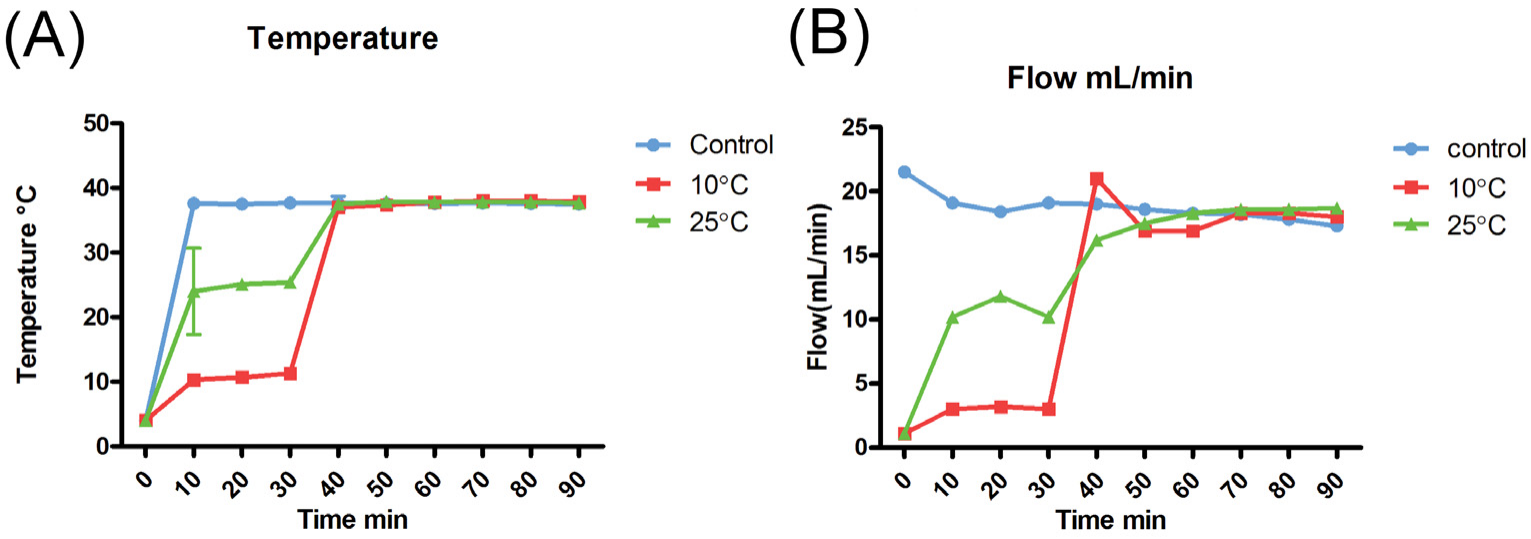
Panel A Thermal variation in the control and gradual rewarming groups during the perfusion period. Values are mean ± standard deviation. Panel B Flow variation in the control group and the rewarming groups during 90 minutes of perfusion. Values are mean ± standard deviation.

Temperature profiles are shown in the “Fig 1, Panel A”. The graph represents the gradual temperature increase in the gradual rewarming groups and the temperature status of the control group during the perfusion time.

Renal flow was gradually increased in the gradual rewarming groups in the first 30 minutes of rewarming. During perfusion and until the end of the perfusion at 38°C there was no difference in flow between the control group and the gradual rewarming groups “Fig 1, Panel B”.

### Functional parameters

**Fig 2:**
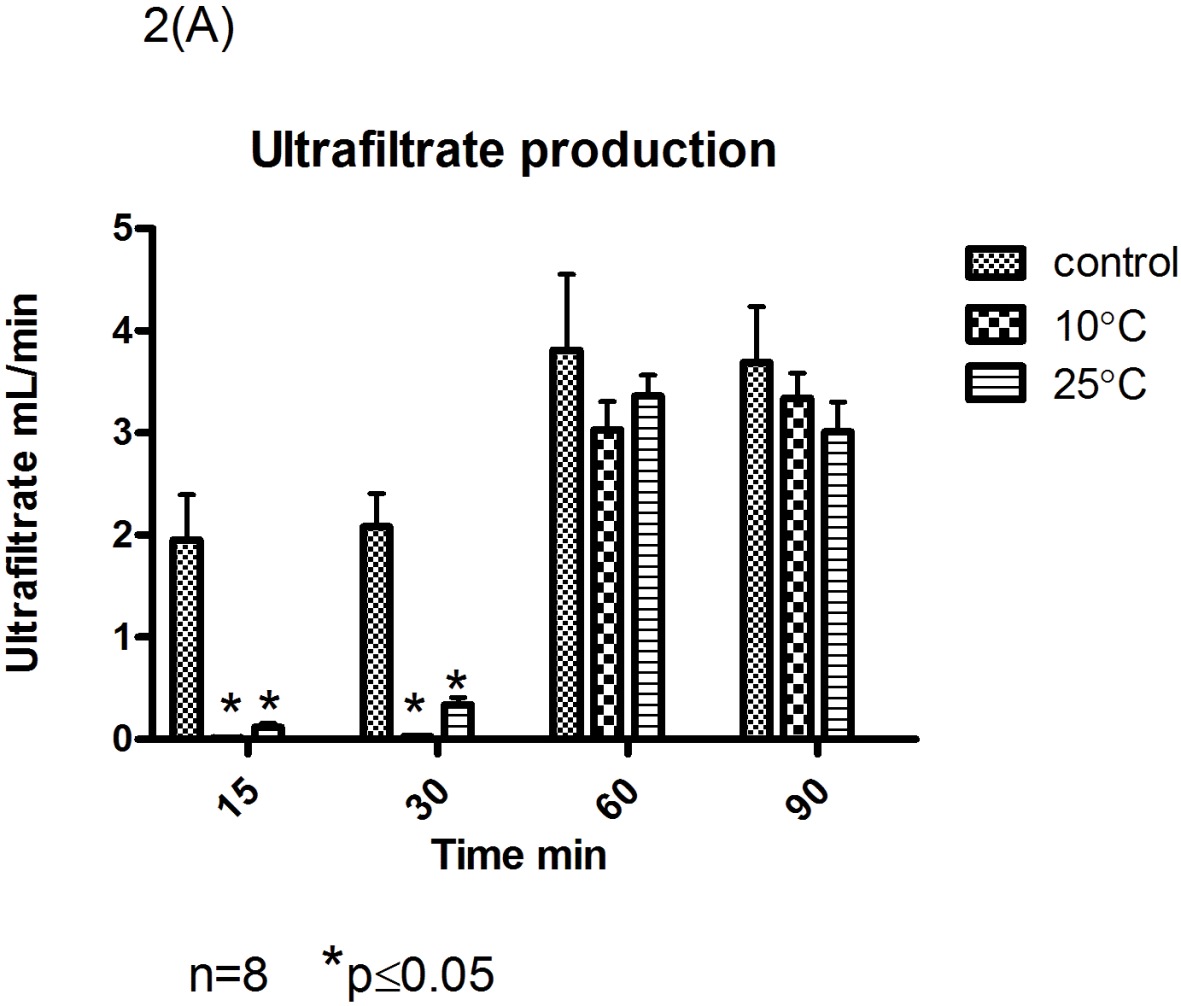
Fig 2 Ultrafiltrate Production at 15, 30, 60 and 90 minutes of the perfusion in the control and gradual rewarming groups. * P<0.05 vs control group. Values are mean ± standard deviation.

Ultrafiltrate production was higher in the control group compared to the gradual rewarming groups “p<0.05; Fig 2”. Fractional re-absorption of sodium however was improved in all the gradual rewarming groups compared to the control group and this, reached statistical significance in the 25°C group at the end of reperfusion (t = 90) “Table 3”. There were no differences in GFR between the control group versus the gradual rewarming groups “Table 3”. After 90 minutes of perfusion there was a significantly lower lactate level in the gradual rewarming groups compared to the control group “Table 3”. In all three groups pH was decreased at the end of the perfusion compared to the beginning of the perfusion “Table 4”.

**Table 3:** Fractional re-absorption of sodium and lactate and LPO level in the perfusate and GFR after 90 minutes of perfusion in the control group and in the gradual rewarming groups. * P<0.05 vs control group. Values are mean ± standard deviation.

in the end of perfusion	control	10°C	25°C	p-value
**Fractional re-absorption of sodium**	29.98±9	42.59±16	46.5±11*	0.005
**GFR**	0.181±0.06	0.202±0.08	0.194±0.08	0.015
**Lactate**	0.8±0.13	0.4±0.05*	0.5±0.04*	P<0.0001
**LPO**	1.05±0.0.8	1.03±0.04	0.9±0.07	0.37

**Table 4:** Acid-base balance in the perfusate at the end of the perfusion period in the control group and the gradual rewarming groups. * P<0.05 vs control group. Values are mean ± standard deviation

pH	Control	10°C	25°C
**Pre-perfusion**	7.41±0.03	7.43±0.03	7.42±0.03
**Post-perfusion**	7.33±0.05	7.17±0.08	7.20±0.05
**p-value**	0.015	P<0.001	P<0.001

### Renal injury biomarkers

**Fig 3:**
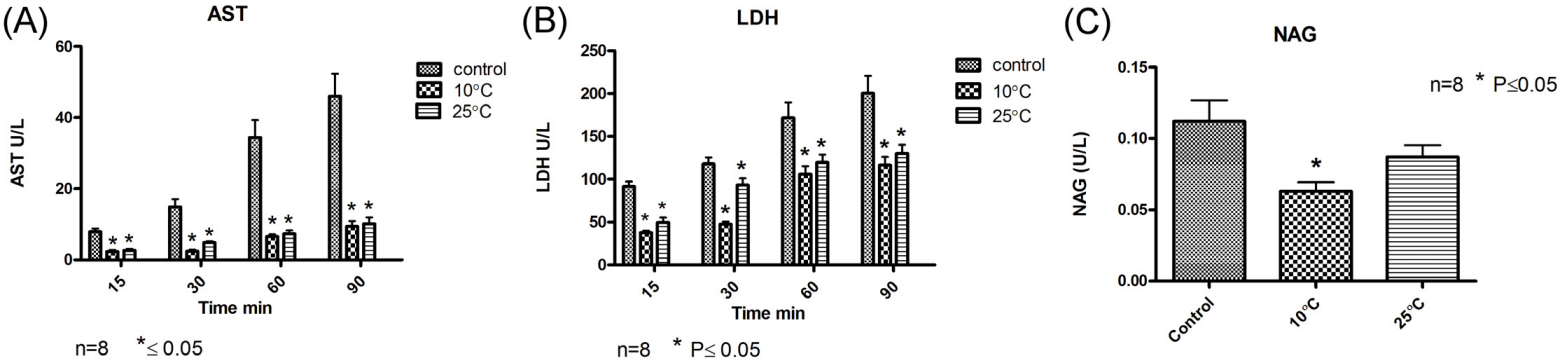
Panel A Perfusate level of AST during 90 minutes of perfusion in the control and gradual rewarming groups. Panel B Perfusate level of LDH during 90 minutes of perfusion. Panel C The level of NAG in the ultrafiltrate during perfusion in the control and gradual rewarming groups. * P<0.05 vs control group. Values are mean ± standard deviation.

Concentrations of AST in the perfusate gradually increased in all four experimental groups during the course of 90 minutes perfusion with the steepest rise observed in the control group versus all gradual rewarming groups “P<0.05; Fig 3, Panel A”. The level of LDH in the perfusate was higher in the control group compared to all gradual rewarming groups during the 60 minutes reperfusion in 38°C “Fig 3, Panel B”. NAG in the ultrafiltrate was lower in the gradual rewarming groups (10°C and slow 38°C) compared to the level of NAG in the control group “P<0.05; Fig 3, Panel C”.

### Lipid peroxidation

The results from LPO measurements in the perfusate samples collected at the end of the perfusion (T = 90 min) showed no statistical difference between the control group and gradual rewarming groups “Table 3”.

### mRNA expression

By the end of perfusion, the level of KIM-1, ICAM-1, VCAM-1 and HSP-70 expression was reduced in the gradual rewarming groups compared to the control group. Also, the expression of P-selectin was numerically reduced in all gradual rewarming groups compared to the control group “Table 5”.

**Table 5:** mRNA expression level of KIM-1 and HSP-70 in the kidney biopsies specified by real-time PCR in the frozen sections from the control group and the gradual rewarming groups. * P<0.05 vs control group. Values are mean ± standard deviation.

mRNA expression	control	10°C	25°C	p-value
**KIM-1**	0.005±0.001	0.002±0.0007*	0.003±0.002*	P≤0.05
**HSP-70**	64.0±12.5	43.6±8.1*	42.2±8.3*	P≤0.05
**ICAM-1**	1.56±0.60	0.74±0.15*	0.97±0.10*	P≤0.05
**VCAM-1**	0.63±0.17	0.29±0.07*	0.34±0.08*	P≤0.05
**P-selectin**	0.18±0.07	0.05±0.02*	0.05±0.02*	P≤0.05

### Energy store—Adenosine 5-triphosphate (ATP)

ATP content was significantly elevated after 90 minutes of perfusion in the control group and gradual rewarming groups in comparison to the cold static preservation group. There was no difference between control group and the gradual rewarming groups “Table 6”.

**Table 6:** Renal ATP content after SCS in the reference, control and the gradual rewarming groups after 90 minutes perfusion in the frozen tissue samples. * P<0.05 vs Reference group. Values are mean ± standard deviation.

	reference	Control	10°C	25°C	p-value
**ATP level**	7±2.4	71±29*	73±9*	70±32*	P<0.0001

### Histology

**Fig 4:**
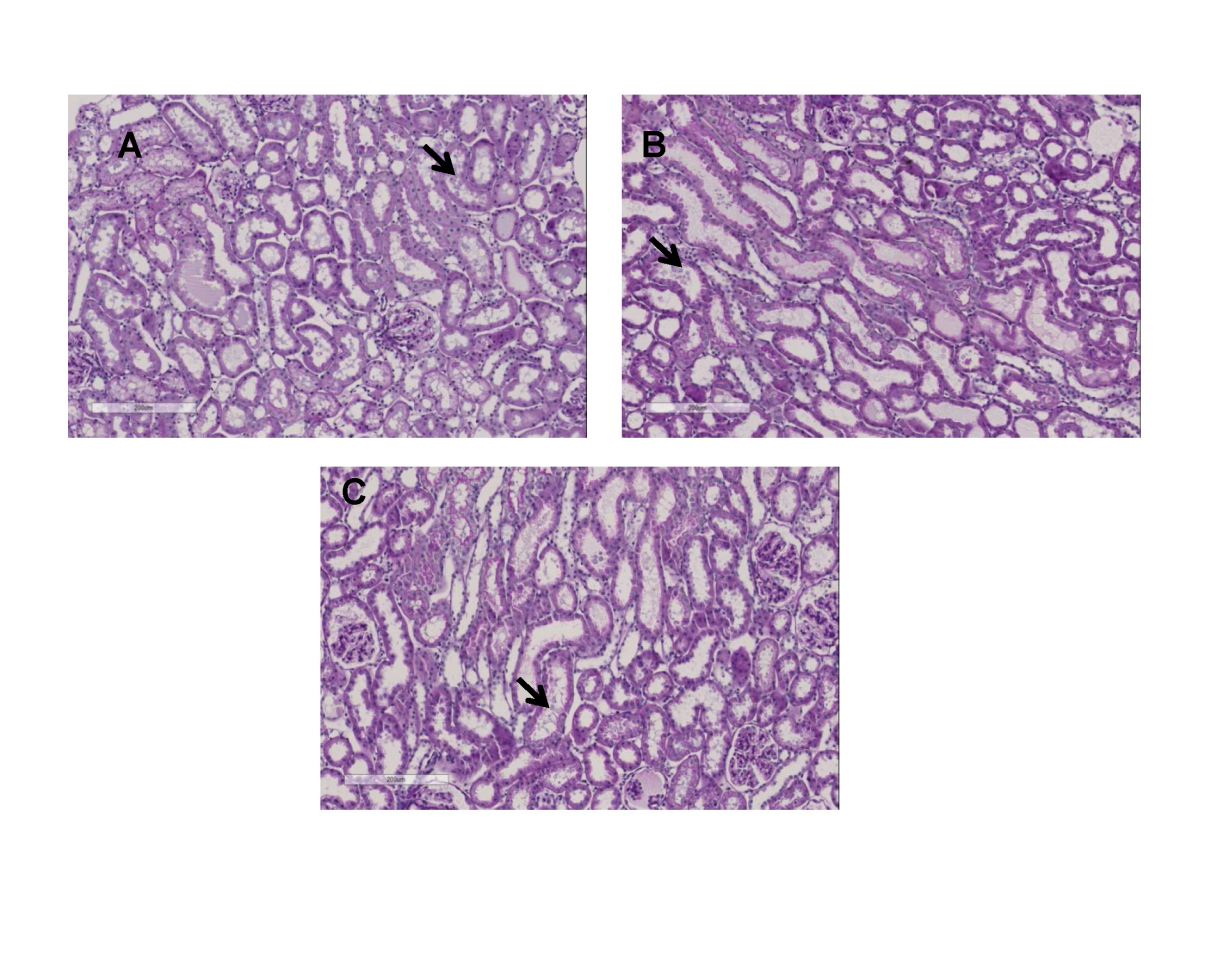
Examples of H&E staining of kidney tissue subjected to: Panel A, immediate rewarming (control group) or gradual rewarming at 10°C Panel B, 25°C Panel C. Epithelial shredding is pointed in all the images.

Light microscopy performed on tissue samples obtained at the end of the experiments did not reveal significant differences among the rewarming groups versus the control group. Overall only slight alterations of normal structural appearance were observed in any group including limited tubular dilation and epithelial shredding “Fig 4”.

## Discussion

Alternations in cellular metabolism and likely cellular injury occur due to energy depletion and accumulation of waste products in an organ during SCS. During graft implantation the re-introduction of warm (37°C) oxygenated blood to the cold (4°C) ischemic organ causes a major release of reactive oxygen species (ROS) and accumulated waste products known as reperfusion injury. Reperfusion injury could result in a delayed graft function and loss of graft viability after transplantation.[18] In liver perfusion, Minor and his colleagues have demonstrated that controlled oxygenated re-warming in an ex-vivo liver perfusion model is correlated with better preservation of liver grafts and improved liver function.[5] Our results are in line with this study as better results were obtained in the gradual rewarming groups.

After reperfusion, lower AST and LDH level in the gradual rewarming groups suggest that the gradual increase in temperature induces less thermal stress that is associated with less parenchymal injury. The results obtained from HSP-70 also support less cellular stress in the gradual rewarming groups. HSP-70 is a heat shock protein which is expressed in the presence of different stress stimuli in cell lines.[19]. Some studies indicate that higher expression of HSP-70 protein is associated with the activation of protective mechanisms.[9] However less expression could also be sign of decreased organ injury.

Acute renal tubular injury is one of the consequences of reperfusion and it might lead to acute kidney failure.[20] It was shown in a study by Han and his colleagues that KIM-1 gene expression as a proximal tubular injury biomarker is undetectable in healthy kidneys. However, the gene is up-regulated after ischemic injury and it is noticeably high after 24–48 hours.[21] Higher gene expression is associated with cellular epithelium differentiation which is the early cellular response to injury. Based on the tubular injury biomarker outcomes such as the lower KIM-1 expression and NAG release, the gradual rewarming strategy used here to reduce tubular injury is promising. The kidneys in the gradual rewarming groups were metabolically more stable as indicated by the lower lactate level which means that these kidneys exert adequate aerobic metabolism.[22] In all three groups a slight acidosis was observed at the end of the perfusion period, this could be resulting from the closed perfusion system used by us, in which the solution was recirculated during the ninety minutes of perfusion.

During kidney perfusion an adequate perfusion pressure is needed in order to support kidney metabolism and to deliver oxygen to the tissue.[23] On the other hand there are some studies showing a correlation between perfusion pressure and endothelial damage due to vascular shear stress.[24,25] Therefore focusing on potential endothelial damage caused by perfusion pressure was another goal of this study. The kidney podocyte cells are very sensitive to shear stress and damage to these cells can lead to organ dysfunction after renal transplant.[26] Shear stress caused by perfusion flow could induce endothelial cellular detachment and subsequently lead to vascular endothelial damage. Damaged endothelial cells play an important role in inflammation and reactive oxygen species (ROS) formation after reperfusion by providing an adhesion cite for inflammatory mediators like monocyte-derived macrophages.[27] In order to maintain vascular integrity and to prevent endothelial damage induced by pressure in the rewarming groups we gradually increased the pressure alongside increasing the temperature. Although there was no difference in flow between the groups during the reperfusion period, kidneys in the control group demonstrated higher endothelial damage indicated by higher expression of I-CAM, V-CAM and P-Selectin.

Kidney ex vivo perfusion is a well-established model to perform different machine perfusion methods in animal study (pig and rat) and even human organs.[9,10,23,28] Oxygenated WME solution was used as perfusion solution in this study. The composition of this solution makes it an applicable candidate as an acellular solution for organ perfusion at normothermic or near normothermic temperature. It has already been shown that warm perfusion with an acellular, nutrient rich solution is helpful to recover from ischemia injury.[29] WME solution used by us in this study has been previously tested as a preservation and perfusion solution and has demonstrated satisfactory results.[6,30] The rationale behind using an oxygenated solution even at lower temperature is based on experimental and even clinical evidence that mitochondrial function and the energy status of the organ during perfusion perfusion can be improved by short term re-oxygenation reducing oxidative stress reaction and further tissue injury both in kidneys and livers.[3,18,31]

Our findings demonstrate that adding an oxygenated perfusion period after SCS is beneficial in improving renal ATP, without the formation of ROS as indicated by the absence of changes in LPO levels measured as an oxidative stress marker. Leducq and colleagues showed that transition in temperature from hypothermia to normothermia is associated with rapid fall of ATP content in the organ and increased mitochondrial permeability.[32]

The main limitation of our study is the short reperfusion time (90 min), which may not be sufficient to see improvement in functional parameters and histological changes between the groups. Although the Isolated Perfused Kidney Model enables the obtainment of multiple samples for assessment of injury prior to transplantation while avoiding the use of recipient animals, the short reperfusion time (90) min is not sufficient to see sustainable improvements in functional parameters and histological changes between the groups. In future studies a transplantation model will be needed to fully investigate the potential of our findings.

In conclusion, our data demonstrate that post SCS gradual rewarming and gradual increase in pressure during perfusion is beneficial in decreasing injury compared to sudden reperfusion at body temperature. Temperature and pressure controlled oxygenated perfusion of kidneys prior to reperfusion could provide a better recovering strategy especially for kidneys at risk for delayed graft function. As the best results were obtained from gradual rewarming from 10 to 38°C future studies demonstrating the potential of this strategy in a relevant transplant model are needed before implementation in the clinical situation.

## Abbreviations

AST: Aspartate Aminotransferase
ATP: Adenosine 5-triphosphate
IPK: Isolated perfused kidney
IRI: Ischemia Reperfusion Injury
LDH: Lactate Dehydrogenase
NMP: Normothermic machine perfusion
PAS: Periodic Acid-Schiff
ROS: Reactive Oxygen Species
SCS: Static cold storage
UW: University of Wisconsin
WME: William’s medium E
