# Correction: Gradual Rewarming with Gradual Increase in Pressure during Machine Perfusion after Cold Static Preservation Reduces Kidney Ischemia Reperfusion Injury

**DOI:** 10.1371/journal.pone.0152006

**Published:** 2016-03-15

**Authors:** Paria Mahboub, Petra Ottens, Marc Seelen, Nils ‘t Hart, Harry Van Goor, Rutger Ploeg, Paulo N Martins, Henri Leuvenink

The fourth and seventh authors’ names are spelled incorrectly. The correct names are: Nils ‘t Hart and Paulo N Martins. The correct citation is: Mahboub P, Ottens P, Seelen M, ‘t Hart N, Van Goor H, Ploeg R, et al. (2015) Gradual Rewarming with Gradual Increase in Pressure during Machine Perfusion after Cold Static Preservation Reduces Kidney Ischemia Reperfusion Injury. PLoS ONE 10(12): e0143859. doi:10.1371/journal.pone.0143859.
